# 2259. Reversal of antimicrobial consumption: long-term impact of COVID-19 pandemic

**DOI:** 10.1093/ofid/ofad500.1881

**Published:** 2023-11-27

**Authors:** Ryuji Koizumi, Shinya Tsuzuki, Yusuke Asai, Norio Ohmagari

**Affiliations:** National Center for Global health and Medicine, Shinjuku-ku, Tokyo, Japan; National Center for Global Health and Medicine, Shinjuku-ku, Tokyo, Japan; National Center for Global Health and Medicine, Shinjuku-ku, Tokyo, Japan; National Centre for Global Health and Medicine, Shinjuku, Tokyo, Japan

## Abstract

**Background:**

The global decrease in antimicrobial consumption (AMC) in 2020 has been associated with the COVID-19 pandemic.

However, it is not clear whether this downward trend will continue.

Several reports, including ESAC-Net, have documented an increase in antimicrobial use after a decrease in antimicrobial consumption following the COVID-19 pandemic in some countries.

The aim of this study is to examine the global trend in AMC following the emergence of COVID-19 in the long term.

**Methods:**

After comparing the change rate of AMC in 67 countries’ using IQVIA MIDAS^®^ monthly sales data of antimicrobials between 2019 and 2020 and between 2021 and 2022, changepoint detection was conducted in time-series data from November 2016 to October 2022.

Additionally, we examined the impact of implementation and lifting of movement restriction in G7 countries by interrupted time-series analysis (ITSA).

**Results:**

In the IQVIA MIDAS sales data, 65 among 67 countries have one or two changepoints (Table1).

59 among 65 countries experienced a decrease in AMC after the beginning of COVID-19 pandemic.

However, 50 among 59 countries showed reversal increase trend of AMC in 2022 (Figure1).

ITSA showed that implementation of movement restriction had negative impact on AMC in all G7 countries (Figure2).

**Table 1.**
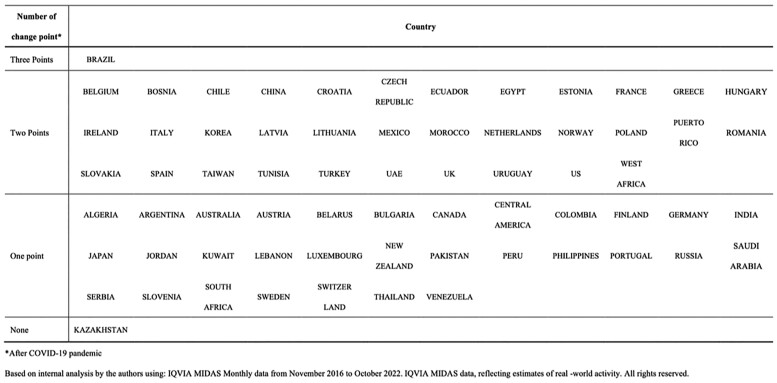
Number of antimicrobial consumption changepoints after COVID-19 pandemic

**Figure 1.**
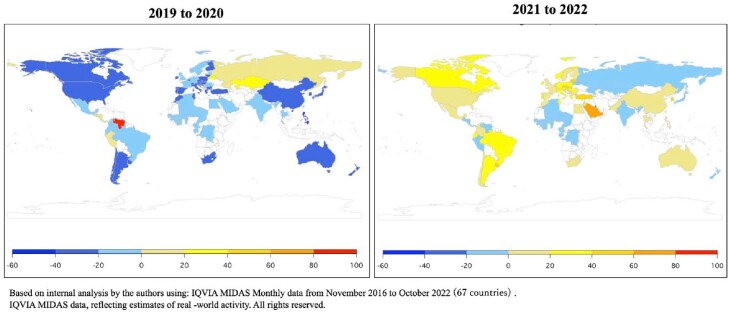
Change rate in monthly antimicrobial consumption in each country from 2019 to 2020 and 2021 to 2022, March-August

**Figure 2.**
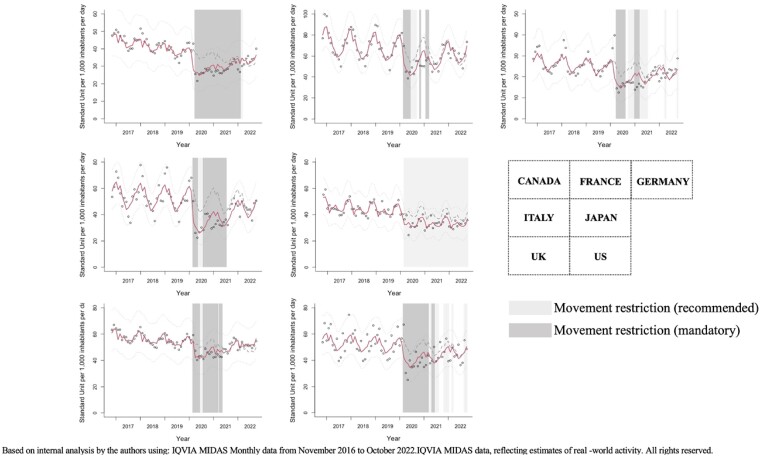
Interrupted time series analysis of antimicrobial consumption data in G7 countries

**Conclusion:**

The global decrease in AMC in 2020 may not be due to COVID-19 itself, but to non-pharmaceutical interventions such as movement restrictions.

Human mobility may be one of the key determinants of antimicrobial use at the population level.

**Disclosures:**

**All Authors**: No reported disclosures

